# Redox Modulation of FAK Controls Melanoma Survival - Role of NOX4

**DOI:** 10.1371/journal.pone.0099481

**Published:** 2014-06-09

**Authors:** Cristiane Ribeiro-Pereira, João Alfredo Moraes, Mariele de Jesus Souza, Francisco R. Laurindo, Maria Augusta Arruda, Christina Barja-Fidalgo

**Affiliations:** 1 Laboratory of Cellular and Molecular Pharmacology, Department of Cell Biology, IBRAG, Universidade do Estado do Rio de Janeiro, Rio de Janeiro, RJ, Brazil; 2 Laboratory of Vascular Biology, Instituto do Coração, Universidade de São Paulo, São Paulo, SP, Brazil; 3 Vice-Diretoria de Ensino, Pesquisa e Inovação, Farmanguinhos, Fiocruz, Rio de Janeiro, RJ, Brazil; University of Illinois at Chicago, United States of America

## Abstract

Studies have demonstrated that reactive oxygen species (ROS) generated by NADPH oxidase are essential for melanoma proliferation and survival. However, the mechanisms by which NADPH oxidase regulates these effects are still unclear. In this work, we investigate the role of NADPH oxidase-derived ROS in the signaling events that coordinate melanoma cell survival. Using the highly metastatic human melanoma cell line MV3, we observed that pharmacological NADPH oxidase inhibition reduced melanoma viability and induced dramatic cellular shape changes. These effects were accompanied by actin cytoskeleton rearrangement, diminished FAK^Y397^ phosphorylation, and decrease of FAK-actin and FAK-cSrc association, indicating disassembly of focal adhesion processes, a phenomenon that often results in anoikis. Accordingly, NADPH oxidase inhibition also enhanced hypodiploid DNA content, and caspase-3 activation, suggesting activation of the apoptotic machinery. NOX4 is likely to be involved in these effects, since silencing of NOX4 significantly inhibited basal ROS production, reduced FAK^Y397^ phosphorylation and decreased tumor cell viability. Altogether, the results suggest that intracellular ROS generated by the NADPH oxidase, most likely NOX4, transmits cell survival signals on melanoma cells through the FAK pathway, maintaining adhesion contacts and cell viability.

## Introduction

Melanoma arises from the malignant transformation of pigment-producing cells (melanocytes), and its incidence has increased in many countries, being a prominent worldwide public health challenge [1]–[3].

 Development of skin cancer is a multistage process mediated by different cellular, biochemical and molecular changes, involving the activation of several anti-apoptotic and pro-survival signaling pathways. Though, a critical step in melanoma biology is its ability to overcome anchorage dependency, acquiring ‘vertical growth phase’ (VGP) properties, enabling these cells to enter the deeper dermis rather than growing only in or adjacent to the epidermis. VGP can therefore make melanoma cells competent to metastasis. The metastatic ability of these cells is closely related to a rearrangement on the integrin-coordinated signaling hierarchy [4], [5].

Epidemiological studies have demonstrated that the major risk factors for melanoma relate to both environmental exposure and genetic alterations. For example, melanoma incidence in white populations has revealed an inverse correlation with latitude and positive correlation with ultraviolet radiation (UVR) index [6]–[7]. Skin exposure to UVR generates ROS in excessive quantities [8]. However, rather than this occurring as a direct effect of UVR, it has been shown that the observed ROS accumulation also relies on ROS generated by highly specialized enzymatic systems [9].

ROS are classically referred as cytotoxic agents due to their ability to oxidize biomolecules [10]. However, the direct cell damage only occurs when their generation is greatly increased and the antioxidant mechanisms are overwhelmed, a condition defined as “oxidative stress” [11]. On the other hand, a growing body of reports shows that rather than being hazardous molecules, ROS are second messengers, able to modulate a number of signaling pathways, many of them involved in tumor development [12], [13].

Among all intracellular ROS-generating systems, the most specialized one is a family of multimeric enzymes called NADPH oxidase [14]. NADPH oxidase was primarily described in neutrophils where they exert a critical role in innate immunity, taking part in the killing of pathogens [15]. NADPH oxidase activity was also detected in other cell types, involving other homologous of the main membrane subunit, NOX. To date, five NOXs have been described (NOX1 – NOX5) [16].

In non-phagocytic cells, NADPH oxidase activity leads to the generation of ROS, which seems to modulate diverse intracellular signaling pathways [17], [18]. While in most cell types NADPH oxidase-dependent ROS generation is triggered and/or stimulated by agonists, many malignant cells constitutively produce ROS in an augmented fashion [19], [20]. Recent works have pointed to a critical role for ROS generated by NADPH oxidase in the initiation and progression phases of malignant cells development [21]–[24]. For example, NOX4 and NOX5 activities have been reported to be involved in the survival of human glioma, melanoma, and prostate cancer cells [25]–[27]. These findings suggest that the modulation of survival and proliferation signaling by ROS plays a critical role in cancer development. However, the mechanisms by which NADPH oxidase regulates these signaling pathways are not fully understood.

In this study, we aimed to characterize the role of endogenously produced ROS in MV3 cells, a highly aggressive human melanoma cell line [28]. Using both pharmacological and gene silencing approaches, we investigated the role of NADPH oxidase activity on cell fate and signaling. Our findings strongly suggest that constitutive NADPH oxidase activity stimulates FAK signaling pathway and protects melanoma cells from death. Furthermore, our data shed light on NOX4 as a novel pharmacological target for the control of melanoma growth and metastasis.

## Material and Methods

### Ethics Statement

Ethical approval was obtained from the Ethics Committee of Pedro Ernesto Hospital, Rio de Janeiro State University (2786/2010) and all volunteers gave written informed consent for participation before enrollment in the study.

### Reagents

HEPES, ethylenediaminetetraacetate (EDTA), bovine serum albumin (BSA), penicillin, streptomycin, dihydrorhodamine 123 (DHR), phenylmethylsulfonyl fluoride (PMSF), benzamidine, leupeptin, and soybean trypsin inhibitor (SBTI), sodium orthovanadate (Na_3_VO_4_), apocynin (4-acetovanillone), 3-(4,5-dimethylthiazol-2-yl)-2-5-diphenol tetrazolium bromide (MTT), pyrrolidine dithiocarbamate (PDTC), diphenyleneiodonium (DPI), TRITC-labelled phalloidin, cycloheximide (CHX), ribonuclease A (RNase A), propidium iodide (PI), diethylenetriamine pentaacetic acid (DTPA), RPMI-1640 medium, superoxide dismutase conjugated to polyethylene glycol (PEG-SOD), catalase conjugated to PEG (PEG-CAT) and sulforhodamine B were from Sigma-Aldrich (St. Louis, MO). Triton X-100, Percoll, PVDF membranes, Rainbow™ were from GE Healthcare (San Francisco, CA). Fetal bovine serum (FBS) was purchased from Cultilab (Campinas, SP, Brazil). Dulbecco's Modified Eagle Medium (DMEM), Dihydroethidium (DHE), anti- p-FAK397 and Lipofectamine 2000 were obtained from Invitrogen (Carlsbad, CA). DAF-FMDA, CM-H2DCFDA, HPF and JC-1 were obtained from Molecular Probes (Carlsbad, CA). siRNA oligomers for NOX4 (5′-CCTCAGCATCTGTTCTTAACCTCAA-3′) and its scrambled sequence (Scramble) were obtained using BLOCKiTTM RNAi Designer (Invitrogen). Antibody anti-caspase-3 was obtained from Cell Signaling. All other antibodies and protein A/G agarose were from Santa Cruz Biotechnology (Santa Cruz, CA). Streptavidin-conjugated FITC and Streptavidin-conjugated horseradish peroxidase were from Caltag Laboratories. ECL system (SuperSignal West Pico chemiluminescent substrate kit) was from Pierce Biotechnology (Rockford, IL, USA). High capacity cDNA reverse transcripition kit RNeasy, RNeasy Mini kit were from Qiagen, RQ1 RNase-Free DNase, the set of dN TP and RNasin RNase inhibitor were purchased from Promega (Madison, WI).

### Cell Culture

MV3 human melanoma cell line [28] was a gift from Dr Cezary Marcienkiewicz (Center for Neurovirology and Cancer Biology, Temple University, PA, USA). MV3 cells were maintained in DMEM enriched with 10% FBS, 3.7 g/L sodium bicarbonate, 5.2 g/L HEPES, 0.5 U/mL penicillin and 0.5 mg/mL streptomycin at 37°C in a humidified atmosphere of 5% CO_2_. Cells were grown to 80-90% confluence into 75 cm culture flasks and were detached by brief treatment with 5 mM EDTA in Hank's balanced salt solution (HBSS).

### Measurement of ROS generation in intact cells by DHR

Intracellular ROS production by MV3 cells was measured by oxidation of dihydrorhodamine 123 (DHR) to rhodamine by H_2_O_2_ as previously described [29]. Briefly, MV3 cells were seeded on 96-well plates at a density of 6×10^3^ cells/well and, after overnight incubation, the medium was replaced by serum-free medium containing DHR (final concentration 50 µM). Cells were then washed with phosphate-buffered saline (PBS) before examination under an Olympus IX71 inverted microscope (Tokyo, Japan) equipped for fluorescence.

### Cellular ROS measurement by HPLC

Ethidium and 2-hydroxyethidium (EOH), the oxidation products of DHE, were separated by HPLC as described above [30]. Briefly, human melanoma (4×10^5^ cells) cells were grown in 6-well dishes in DMEM medium supplemented with fetal bovine serum (10%). After adhesion, melanoma cells were incubated with DPI (10 µM) or SOD conjugated to polyethylene glycol (PEG-SOD, 25 U/mL), catalase conjugated to PEG (PEG-CAT, 200 U/mL), or both PEG-CAT (200 U/mL) and PEG-SOD (25 U/mL) for 1 hour at 37°C in a 5% CO_2_ atmosphere. Cells were washed twice with Krebs (0.5 mM CaCl_2_, 1.2 mM MgSO_4_, 4.9 mM KCl, 5.7 KH_2_PO_4_, 145 mM NaCl, 5.7 mM Na_2_HPO_4_ and glucose 5.5 mM pH 7.4) and incubated in Krebs, containing DTPA (100 µM) at a final DHE concentration of 50 µM for additional 30 min. Cells were washed with Krebs, harvested in acetonitrile (0.5 mL/well) and centrifuged (12,000 g for 10 min at 4°C). Supernatants were dried under vacuum (Speed Vac Plus model SC-110A, Thermo Savant) and pellets maintained at −70°C in the dark until analysis. Samples were ressuspended in 60 µL in solution A (water/10% acetonitrile/0.1% trifluoracetic acid) and injected (50 µL) into HPLC system.

### Real-time intracellular ROS production

MV3 melanoma cells (and MV3 cells silenced with siRNA scramble or siRNA NOX4, when mentioned) were detached by brief treatment with 5 mM EDTA in HBSS, collected by centrifugation, ressuspended in fresh 10% FBS DMEM medium and incubated overnight on 96-well black plates at a density of 6×10^3^ cells/well, at 37°C in a humidified atmosphere of 5% CO_2_. The cells were washed three times with PBS, incubated in HBSS at 37°C, 5% CO_2_ for 1 h. Cells were then loaded for 1 h with one of the intracellular ROS detection probes depicted below (final concentration 5 µM). Once loaded, cells were washed with PBS and treated or not with DPI. Fluorescence intensity was assessed throughout 3 h using in the microplate reader Envision™.

#### DAF-FM DA assay

NO production was assessed by the fluorescence emitted by oxidized DAF, an specific probe for NO detection. Fluorescence was monitored at excitation and emission wavelengths of 495 nm and 515 nm, respectively.

#### CM-H2DCF DA assay

ROS production was detected through fluorescence emitted from DCF oxidation. This probe detects ROS in general, being more selective to H2O2 and peroxynitrite. Fluorescence was monitored at excitation and emission wavelengths of 495 nm and 525 nm, respectively.

#### HPF assay

Peroxynitrite (ONOO-) production was determined by monitoring the fluorescence resulted from HPF oxidation. This probe, which is selective for ONOO-, had its fluorescence detected at excitation and emission wavelengths of 490 nm and 515 nm, respectively.

### Cell viability assays

#### MTT assay

MV3 melanoma cells were detached by brief treatment with 5 mM EDTA in HBSS, collected by centrifugation, ressuspended in fresh 10% FBS medium DMEM and placed into 96-well plates at a density of 6×10^3^ cells/well. After adhesion, cells were incubated in the presence or absence of DPI (0.1–10 µM), Na_3_VO_4_ (0.1–3 µM), apocynin (1–10 µM) or PDTC (0.1–1 µM), at 37°C in humidified 5% CO_2_. After 48 hours of incubation or after 48, 72 or 96 hours of siRNA transfection, MTT assay was performed as previously described [31]. Cells were incubated with MTT (50 µg/well) in the dark at 37°C for 4 hours, when MTT is reduced to formazan crystals by viable cells. After incubation, the formazan crystals were dissolved in isoprapanol and the optical densitometry obtained using a microplate reader (BIO-RAD) using a 570 nM filter. Results are shown as percentage of control, of three independent experiments performed in quintuplicate.

#### Sulforhodamine B assay

MV3 melanoma cells were detached by brief treatment with 5 mM EDTA in HBSS, collected by centrifugation, ressuspended in fresh 10% FBS medium DMEM and placed into 96-well plates at a density of 6×10^3^ cells/well. After adhesion, cells were incubated in the presence or absence of DPI (10 µM), apocynin (10 µM) or cycloheximide (5 µM), at 37°C in humidified 5% CO_2_. After 48 hours of incubation, or after 48, 72 or 96 hours of siRNA transfection, the medium was removed, and cells were fixed with cold 10% trichloracetic acid (TCA) for 1 hour at 4°C. Plates were washed 5 times with Milli-Q water and left to dry at room temperature. Cells were stained with 0.4% of sulforhodamine B (w/v) in 1% acetic acid (v/v) at room temperature for 10 minutes. sulforhodamine B was removed, and the plates were washed 5× with 1% acetic acid before air-drying. Bound dye was solubilized with 10 mM unbuffered Tris-base solution, and plates were left on a plate shaker for at least 10 minutes. Absorbance was measured in a 96-well plate reader (BIO-RAD), at 490 nm.

### Immunocytochemistry and cytochemistry assays

For immunofluorescence microscopy, MV3 cells were grown on glass coverslips (9.0×10^3^ cells/well). After adhesion, MV3 melanoma cells were incubated in the presence or absence of DPI (10 µM) for 30 min, 2 and 4 h at 37°C and 5% CO_2_ atmosphere. Following treatments, cells were washed with ice-cold PBS and fixed with PBS containing 4% paraformaldehyde/4% sucrose for 20 min at room temperature and permeabilized in PBS containing 0.2% Triton X-100 for 5 min. Then, cells were incubated with PBS-BSA for 30 min. For detection of FAK, permeabilized cells were incubated with anti-FAK antibody (1∶200) overnight at 4°C and then sequentially incubated with goat anti-IgG Ab biotin-conjugated for 1 h and streptavidin-conjugated FITC (1∶200) for 1 h. Filamentous actin was stained with rhodamin-conjugated phalloidin (1∶1000) for 2 h at room temperature. Coverlips were mounted onto microscope slides using a solution of 20 mM N-propylgallate and 20% glycerol in PBS. Microscopic analysis of FAK- and phalloidin-stained cells were carried out using a laser scanning confocal microscope (Olympus - Fluoview version 3.3).

### Apoptosis detection

#### Hypodiploid DNA content analysis

MV3 cells were detached by brief treatment with 5 mM EDTA in HBSS, collected by centrifugation and placed into 6-well plates at a density of 5×10^5^ cells/well in 10% FBS medium. After adhesion, melanoma cells were incubated in the presence or absence of DPI (10 µM), apocynin (10 µM) or cycloheximide (5 µM) for 24 h. Thereafter, cells detached with 5 mM EDTA in HBSS and collected by centrifugation were ressuspended in PBS, fixed with ethanol 70% for 2 h and incubated in PBS solution containing 200 µg/mL RNase A, 0.001% Triton X-100, 20 µg/mL PI for 30 min at room temperature. DNA contents in stained nuclei were analyzed with FACScan (Becton Dickinson). A suspension of cells was analyzed for each DNA histogram, and the percentage of cells in G0/G1, S, and G2/M phases was determined using the WINMDI program.

### Experiments using RNA interference

NOX4 gene expression was repressed using RNA interference technology according to Lipofectamine 2000 manufacturer's protocol. MV3 cells were seeded in 6-well (2.5×10^5^/well, for western blotting or qRTPCR) or 96-well (5×10^3^, for ROS production kinectics analysis, MTT, sulforhodamine B and JC-1 assay plates. The transfection efficiency was determined with qRT-PCR and Western blotting.

### Assessment of mitochondrial transmembrane potential (JC-1)

The mitochondrial stability was measured by the use of the cationic dye JC-1, which is incorporated to the mitochondrial intermembrane space. The monomer (green) can polymerize forming clusters known as J-aggregates (red) in a transmembrane potential-dependent manner. Therefore, viable, non-apoptotic cells exhibit a higher red/green ratio [32]. MV3 melanoma cells were seeded overnight into 96-well black plates at a density of 5×10^3^ cells/well. Cells were then, treated for 24 h with scramble siRNA or NOX4 siRNA. After that, cells were washed with PBS and stained with JC-1 (10 µg/mL) for 30 min. Mitochondrial transmembrane potential was monitored using Envision™ multilable plate reader. Red fluorescence intensity was assessed by excitation and emission at 560 nm and 595 nm, respectively and green fluorescence intensity was detected by excitation and emission at 485 nm and 535 nm, respectively. Mitochondrial transmembrane potential was expressed as the ratio red/green fluorescence.

### Total cell extracts

After the incubation, the cells were suspended in lysis buffer (HEPES 20 mM, pH 7.9; glicerol 20% (v/v); NP-40 1% (v/v); MgCl_2_ 1 mM; EDTA 0.5 mM; EGTA 0.1 mM; DTT 0.5 mM, Na_3_VO_4_ 1 mM) and the following protease inhibitors: PMSF (1 mM), aprotinin (2 µg/mL), leupeptin (2 µg/mL) and SBTI (2 µg/mL).

### Immunoprecipitation

After adhesion, MV3 cells (5×10^5^ cells) were incubated in the presence or absence of DPI (10 µM) for 2 and 4 h at 37°C in a 5% CO_2_ atmosphere. The cells were lysed with RIPA buffer (50 mM Tris-HCl, pH 8.0, 150 mM NaCl, 1% Sodium Desoxicolate w/v, EDTA 5 mM; 1% Triton X-100 v/v, 50 mM NaF, 1 mM Na_3_VO_4_, 30 mM Na_4_P_2_O_7_, 10 mM Iodocetamide, 2 mM DTT) supplemented with the following protease inhibitors: PMSF (1 mM), aprotinin (2 µg/mL), leupeptin (2 µg/mL) and SBTI (2 µg/mL). Lysates were incubated for 2 h with polyclonal anti-FAK (1∶200) at 4°C in a rotatory shaker. After this time, protein A/G agarose (20 µL/mg protein) was added, and the samples were incubated overnight at 4°C. The content of FAK and cSrc were analyzed by Western Blotting, as described below.

### Immunoblotting analysis

The total protein content in cell extracts was determined by BCA kit. Cell lysates were denatured in sample buffer (50 mM Tris-HCl, pH 6.8, 1% SDS, 5% 2-mercaptoethanol, 10% glycerol, 0.001% bromophenol blue) and heated in a boiling water bath for 3 min. Samples (30 µg total protein) were resolved in 12% or 10% SDS-PAGE and proteins were transferred to PVDF membranes. Rainbow™ colored protein molecular weight markers were run in parallel in order to estimate molecular weights. Membranes were blocked with Tween-TBS (20 mM Tris, 0.5 M NaCl, pH 7.5, 0.1% Tween-20) containing 5% bovine serum albumin (BSA). Primary antibodies used in Western analysis were anti-FAK (1∶500); anti-phospho-FAK397 (1∶500), anti-caspase-3 (1∶500), anti-cSrc (1∶1000), anti-NOX4 (1∶1000), anti-NOX2 (anti-gp91phox; 1∶1000), anti-p22phox (1∶1000), anti-p40phox (1∶1000), anti-p47phox (1∶1000), anti-p67phox (1∶1000) and anti-βTubulin (1∶1000). The PVDF sheets were washed three times with Tween-PBS, followed by 1 h incubation with appropriate secondary antibody conjugated to biotin. Then, PVDF sheets were incubated with streptavidin-conjugated horseradish peroxidase (1∶10000) for 1 h and developed by an ECL system. The bands were quantified by densitometry, using Scion Image Software (Scion Co, Frederick, Maryland, USA).

### Isolation of human neutrophils

Human neutrophils were isolated from 0.5% EDTA treated peripheral venous blood of healthy volunteers, using a four-step discontinuous Percoll gradient [33]. Erythrocytes were removed by hypotonic lysis. Isolated neutrophils (98% purity), estimated to be at least 96% viable by trypan blue dye exclusion, were ressuspended in RPMI-1640 medium.

### RNA isolation and RT-PCR

Total RNA from MV3 melanoma cells (5×10^5^ cells), NGM melanocytes (were obtained by cell bank of Rio de Janeiro) (5×10^5^ cells) and human neutrophils (2×10^6^ cells) were isolated using RNeasy Mini kit. After DNase treatment (RQ1 RNase-Free DNase), the mRNA was reverse transcribed using high capacity cDNA reverse transcripition kit. Primers based on the sequence of human p47phox (GeneBank accession n° NM_000265). The following primers were used to amplify p47phox cDNA: sense, 5′ – ATGAGCCTGCCCACCAAGAT - 3′ (374–393), and antisense, 5′ – TCGAGGAAGGATGCTCCCAT - 3′ (683–702). The expected size of the p47phox PCR product was 328 bp. PCR was performed with the following parameters: 95°C for 5 min for 1 cycle and 32 cycles of denaturation at 95°C for 45 s, annealing at 58°C for 30 s, and elongation at 72°C for 30 s. The following primers were used to amplify NOX4 cDNA: sense, 5′ – TCACAGAAGGTTCCAAGCAG - 3′ (491–510), and antisense, 5′- CTGTATTTTCTCAGGCGTGC - 3′ (571–590). The expected size of the NOX4 PCR product was 91 bp. PCR was performed with the following parameters: One cycle of 95°C for 3 min followed by 35 cycles of denaturation at 95°C for 45 s, annealing at 59°C for 45 s, and elongation at 72°C for 30 s. GAPDH primers were used to validate the cDNA in each reaction. PCR products were separated by 2% agarose gel electrophoresis and visualized by UV exposure on transilluminator.

For qPCR assay the PCR products were obtained using a GeneAmo PCR System 2400 (Perkin Elmer). The Quantitative real time PCR was performed in a Rotor gene Q using a SYBR-green fluorescence quantification system (Qiagen) to quantify amplicons. The standard PCR conditions were 95° for 5 minutes, then 35 cycles at 95°C (5 s) and 60°C (10 s) followed by the standard denaturation curve. Before normalizing the values we performed ΔΔCT in function of actin gene expression.

### Statistical analysis

Statistical significance was assessed by the two-tailed unpaired Student's t test. Data were log-transformed when required. Differences were considered statistically significant when p≤0.05. The data were analyzed using GraphPad Prism version 5.00 for Windows (GraphPad Software, USA).

## Results

### Constitutive ROS generation by MV3 melanoma cells requires NADPH oxidase activity

**Figure 1:**
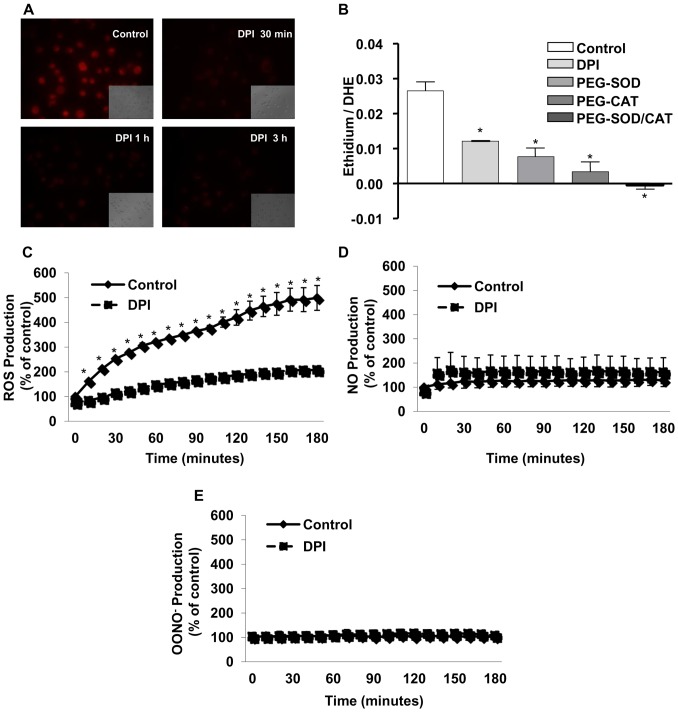
Inhibition of NADPH oxidase activity abolishes intracellular ROS generation on melanoma cells MV3. (A) After adhesion, melanoma cells were incubated with or without DPI (10 µM) for different times (0.5–3 h) and ROS generation was evaluated by dihydrorhodamine-123 (DHR) assay followed by fluorescence microscopy analysis. (B) MV3 cells were incubated for 1 h with DPI (10 µM), PEG-SOD (25 U/mL), PEG-CAT (200 U/mL), or PEG-CAT and PEG-SOD (200 U/mL, 25 U/mL, respectively). Cellular ROS production was measured by intracellular oxidation of DHE to ethidium assessed by HPLC. (C–E) MV3 cells were incubated with or without DPI (10 µM). Intracellular ROS production was measured by intracellular oxidation of CM-H2DCFDA (C), DAF-AM (D) or HPF (E), as described in Material and Methods. Data are expressed as mean ± SD of three independent experiments. * p<0.05 vs. control;

It has already been described that some melanoma cell lines can produce intracellular ROS in a NADPH oxidase-dependent manner [26]. We have observed, for the first time constitutive intracellular ROS generation by the human melanoma cell line MV3, using the dihydrorhodamine (DHR) assay. The non-fluorescent DHR is oxidized to the fluorescent rhodamine, indicating an intracellular accumulation of ROS (Fig. 1A). The fluorescence intensity dramatically diminished when cells were pre-incubated with the flavoprotein inhibitor DPI (10 µM; Fig. 1A), which selectively inhibits NADPH oxidase activity in the concentration range used in this study. Additionally, we have also assessed intracellular ROS generation monitoring dihydroethidium (DHE) conversion to ethidium. Results shown in Figure 1B suggest that superoxide and hydrogen peroxide are the major reactive oxygen species produced by MV3 cells, since the treatment with SOD and catalase impaired ethidium accumulation. Furthermore, the inhibition by DPI confirms that ROS generation by MV3 cells depends on NADPH oxidase activation (Fig. 1B).

In order to determine which ROS are constitutively produced by MV3 cells, we employed selective probes for different reactive oxygen and nitrogen species. MV3 cells constitutively produce considerable amounts of ROS, in a NADPH oxidase-dependent manner (Fig. 1C) and low amounts NO, which was DPI-insensitive (Fig. 1D). No detectable amounts of ONOO^−^ were generated by MV3 cells in basal conditions (Fig. 1E). These results strongly suggest that NADPH oxidase activity is the major source of ROS in resting MV3 melanoma cells.

### NADPH oxidase-derived ROS are involved in MV3 melanoma cells survival

**Figure 2:**
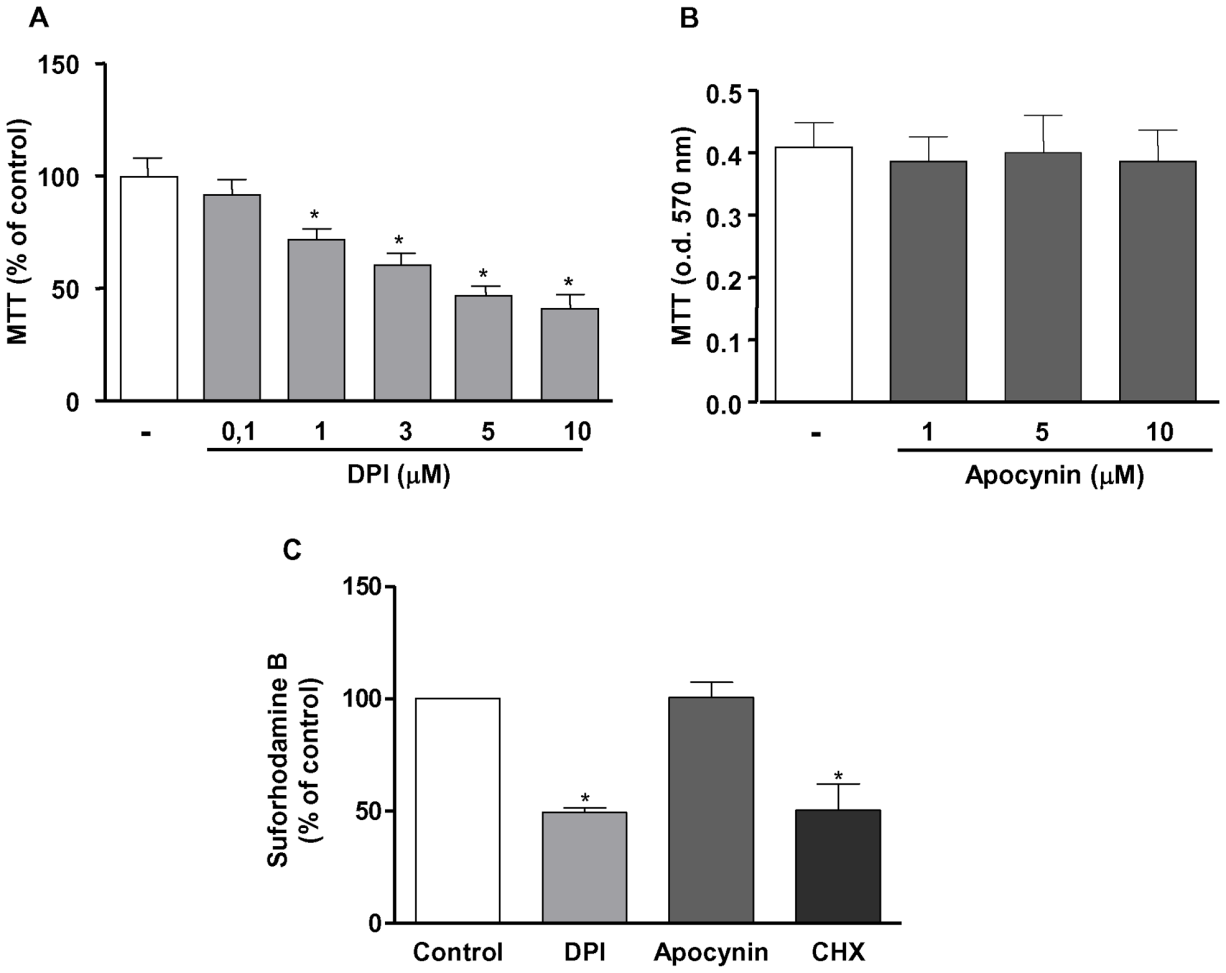
Inhibition of NADPH oxidase activity reduces survival of human melanoma cells MV3. (A–B) MV3 cells (6×10^3^) were incubated for 48 h with or without DPI (A; 0.1–10 µM) or apocynin (B; 1–10 µM), and MTT assay was performed as indicated in Material and Methods. (C) MV3 cells (6×10^3^) were incubated for 48 h with or without DPI (10 µM), apocynin (10 µM) or Cycloheximide (5 µM). Subsequently, sulforhodamine-B assay was performed as described in Materials and Methods. Results are shown as percentage of control and expressed as mean ± SD of at least three independent experiments performed in quintuplicate. *p<0.05 vs. control.

**Figure 3:**
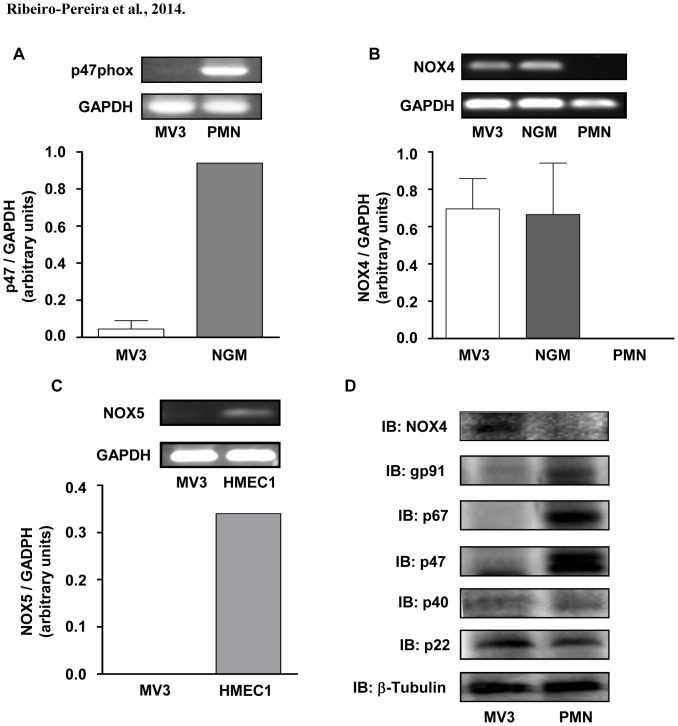
Human melanoma cells MV3 express NOX4, but not NOX5 or NOX2-related NADPH oxidase subunits. Total RNAs were extracted from MV3 melanoma cells and from human polymorphonuclear neutrophils (A–B, PMN), human melanocytes (B, NGM) or from a human microvasculature endothelial cells linage (C, HMEC-1), used as positive controls. The expression levels of p47phox (A), NOX4 (B) or NOX5 (C) were analyzed by RT-PCR, using GAPDH expression as internal control as described in Materials and Methods. (D) NOX4, NOX2, p22phox, p40phox, p47phox, p67phox and βTubulin protein expression were detected by western blotting as described in Materials and Methods. Data are expressed as mean ± SD of three independent experiments and images are representative of three independent experiments with similar results.

As observed in other melanoma cell lines [26], MV3 melanoma cells are highly sensitive to NADPH oxidase inhibition. DPI inhibits cell survival in a concentration-dependent manner, as assessed by MTT (Fig. 2A) and Sulforhodamine B (Fig. 2C) assays. However, apocynin, an inhibitor of the cytosolic subunit p47phox coupling to NOX2 [34] has no effect on MV3 survival (Fig. 2B and 2C), indicating that the production of ROS by MV3 cells is not related to NOX2 activity. Confirming the irrelevance of NOX2 in these effects, MV3 cells do not express p47phox, which is essential to NOX2 activity (Fig. 3A). We have also observed that MV3 cells express high levels of NOX4 mRNA (Fig. 3B). Furthermore, these melanoma cells do not express NOX5, another NADPH oxidase isoform, which does not depend on any of the classical cytosolic NADPH oxidase subunits and is present in endothelial cells (Fig. 3C). We also analyzed NOX subunits expression and we confirmed that MV3 expresses negligible levels of p40phox, p47phox, p67phox and NOX2 (components of the NOX2 NADPH oxidase complex). On the other hand, MV3 expresses NOX4 and p22phox (Fig. 3D). These results indicated that neither NOX2 nor NOX5 contribute for ROS production and strongly suggest that NOX4 is probably the major source of endogenous ROS in MV3 melanoma cell line.

### NADPH oxidase regulates focal adhesions and actin cytoskeleton dynamics in MV3 melanoma cells

We had observed, during routine cell culture monitoring, that melanoma cells treated with DPI displayed severe morphological alterations in early time points, without affecting cell integrity (data not shown). We therefore investigated whether NADPH oxidase-derived ROS could play a role on actin cytoskeleton dynamics and focal adhesion stability in MV3 cells.

**Figure 4:**
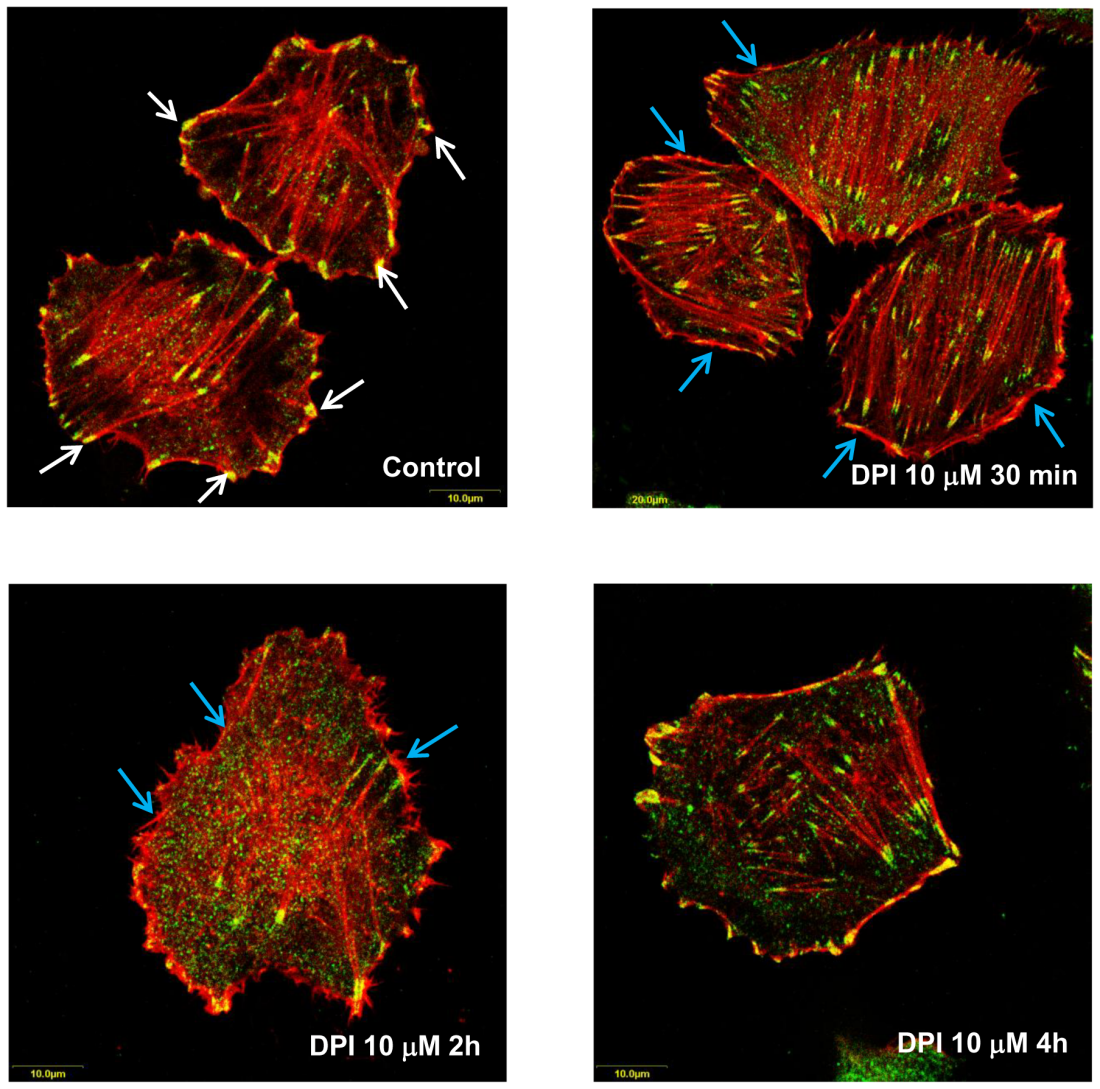
NADPH oxidase regulates cytoskeleton dynamics and focal adhesion sites. MV3 adherent cells were incubated with DPI (10 µM) for 0.5, 2 and 4 hours and double-stained for FAK (FITC, green) and F-actin (TRITC-phalloidin, red). Co-localization was recognized as yellow dots, as indicated by white arrows and F-actin cortical distribution indicated by blue arrows. Images were obtained with a laser scanning confocal microscope (100X). Images are representative of three independent experiments with similar results.

In control cell cultures filamentous actin (red) and FAK (green) can be found co-localized at the cell border as seen by the formation of false yellow dots (white arrows), conferring cell adhesion and stability (Fig. 4; control). The treatment with DPI (10 µM) reduced cell spreading and promoted actin network rearrangement since very early time points (Fig.4; 30 min). The yellow dots, representing actin and FAK co-localization, are no longer seen homogenously distributed along the cell edges after 30 min incubation with DPI, being more abundantly found in the cytosol, associated to the ends of actin stress fibers, which do not reach the periphery of the cell body. At extended incubation times (2h) actin assumes a cortical arrangement (blue arrows), FAK is dispersed in cytosol (green fluorescence), and there is a reduced number of focal adhesions.

After 4 h treatment, cells seem to be irreversibly committed to undergo apoptosis, presenting dramatic disruption of the actin cytoskeleton organization, which is accompanied by alterations in the normal cellular distribution of FAK. At this time point, FAK does not localize in focal adhesion-like structures, but abnormally accumulates at non-specialized sites of the cell membrane. Those alterations precede cell detachment and consequent cell death.

### FAK^Y397^ phosphorylation and its association to cSrc requires NADPH oxidase activity

**Figure 5:**
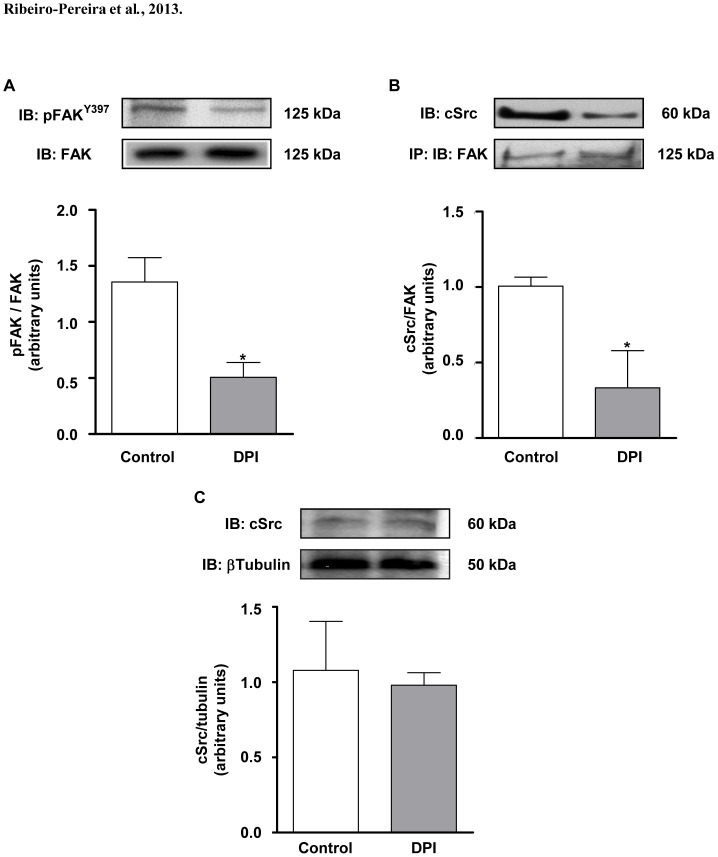
Inhibition of NADPH oxidase activity reduces FAK^Y397^ phosphorylation and FAK-Src association on human melanoma cells. (A) DPI (10 µM) was added to adherent cells (MV3) for 2 hours. Afterwards, total extracts obtained and the content of FAK and FAK^Y397^ was assessed by immunoblotting. Blots were analyzed by densitometry, and phospho-FAK^Y397^/total FAK ratio content is expressed as arbitrary units. (B) After adhesion MV3 cells were incubated in the presence or absence of DPI (10 µM) for 2 hours. Cells were then harvested, lysed and cell extracts were immunoprecipitated with anti-FAK and Western blots were performed for FAK and cSrc detection. Blots were analyzed by densitometry, and cSrc/total FAK ratio content was expressed as arbitrary units. (C) DPI (10 µM) was added to adherent cells (MV3) for 2 hours. Afterwards, total extracts obtained and the content of cSrc and βTubulin was assessed by immunoblotting. Blots were analyzed by densitometry, and cSrc/βTubulin ratio content is expressed as arbitrary units. *p<0.05 vs. control. Images are representative of three independent experiments.

Early studies have demonstrated that ROS can modulate integrin-mediated signaling, enhancing FAK autophosphorylation at Tyr397 [35], an essential step to FAK assembly to polymerized actin. Western blotting analysis showed that NADPH oxidase inhibition by DPI drastically reduces FAK phosphorylation (Fig. 5A). Moreover, treatment with DPI significantly reduced FAK-cSrc association (Figure 5B) without changing cSrc expression (Figure 5C).

### NADPH oxidase inhibition induces apoptosis in melanoma cells

**Figure 6:**
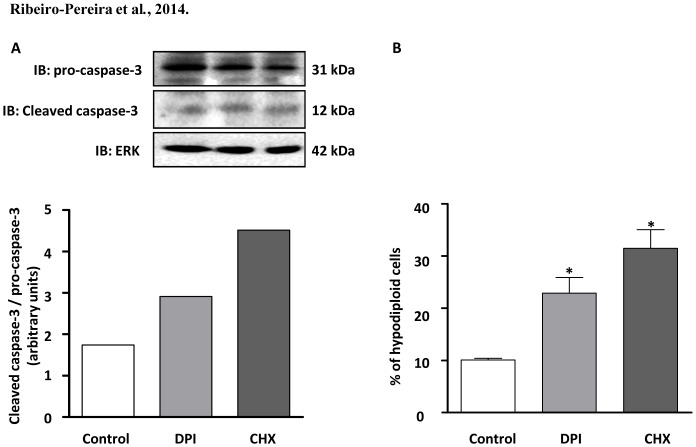
Inhibition of NADPH oxidase activity induces apoptosis on human melanoma cells. (A) After adhesion, MV3 cells were incubated in the presence or absence DPI (10 µM) or CHX (5 µM) for 18 h. Cells were lysed and cell extracts submitted to SDS-PAGE in order to detect procaspase-3 and cleaved caspases-3 content, which were then analyzed by densitometry. ERK served as a loading control. Data shown are representative of three independent experiments with similar results. (B) After adhesion, cells were incubated in the absence or in the presence of DPI (10 µM) or CHX (5 µM) for 24 h. Cells were then harvested, fixed and stained with PI. Flow cytometric analysis of DNA contents was determined using WINMDI software. Percentage of apoptotic (in sub-G0) cells are expressed as mean ± SD of three independent experiments. *p<0.05 vs. control.

Treatment of MV3 melanoma cells with DPI induced procaspase-3 cleavage, a critical step to the generation of active caspase-3 (Fig. 6A). Cell cycle analysis performed with PI-stained cells shows that DPI significantly increased the percentage of hypodiploid cells, indicating apoptosis (Fig. 6B). DPI effect was comparable to the classical inductor of apoptosis, the protein synthesis inhibitor, cycloheximide. As MV3 melanoma cells, constitutively presents high expression of phosphatidylserine on outer cell membrane, it was not possible to detect cell apoptosis through fluorescent Annexin-V binding assay (data not shown).

### NOX4 silencing diminishes phosphorylated FAK content and MV3 cell survival

**Figure 7:**
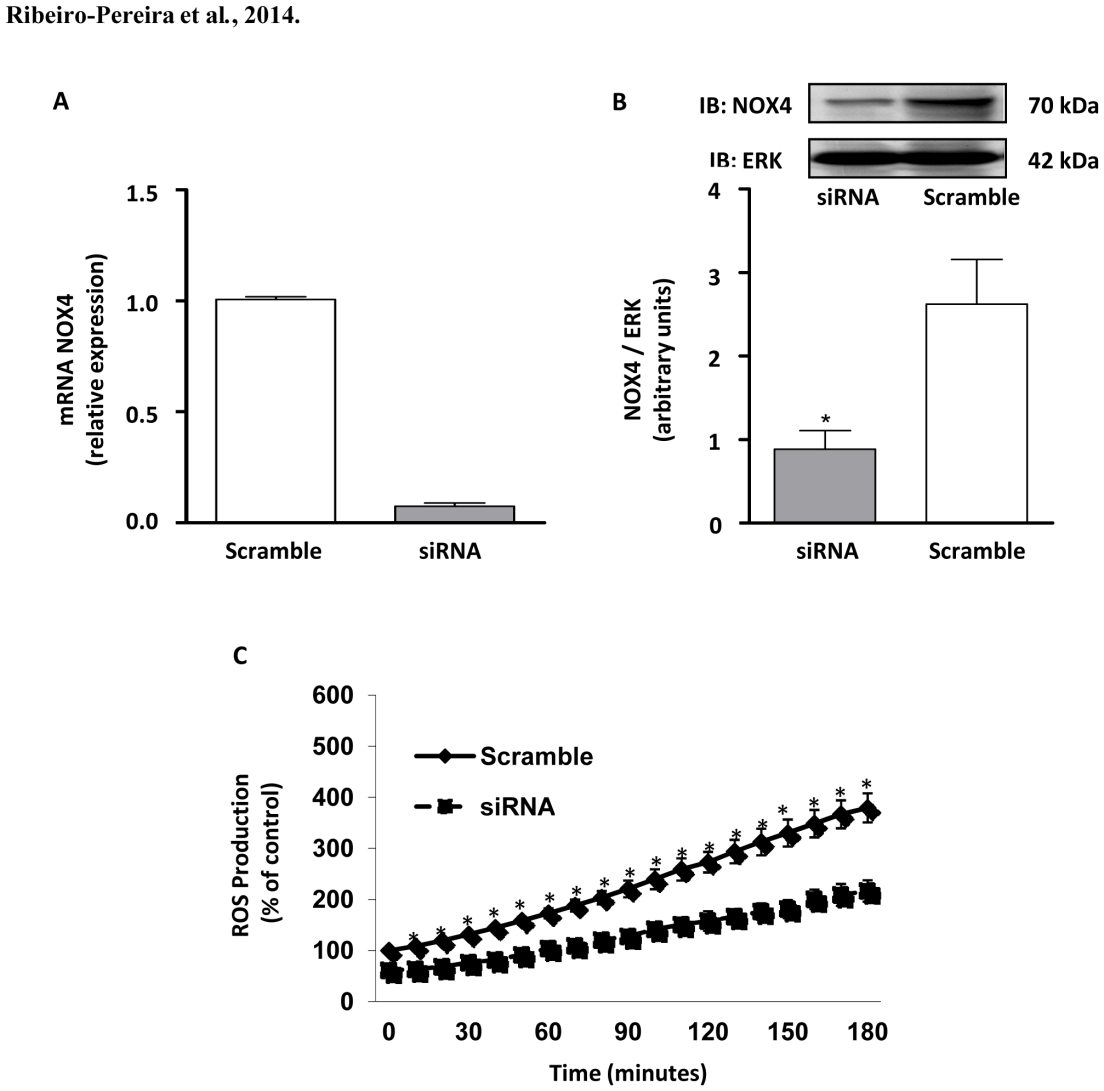
NOX4 silencing reduces MV3 melanoma basal ROS production. (A) Total mRNAs were extracted from the MV3 cells carrying scramble siRNA (Sc) or NOX4-specific siRNA (siRNA) and qRT-PCR was performed to analyze NOX4 mRNA expression, with actin as internal control as described in Materials and Methods. (B) Lysates obtained from transfected cells (10^6^ cells) were subjected to immunoblotting to detect NOX4. Data are expressed as mean ± SD of three independent experiments and images are representative of three independent experiments with similar results. *p<0.05 vs. Scramble. (C) ROS detection assay (CM-H_2_DCFDA) was performed with MV3 melanoma cells transfected with Scramble or NOX4 siRNA, as described in Materials and Methods. Results are shown as percentage of Scramble of three independent experiments *p<0.05 vs. Scramble.

**Figure 8:**
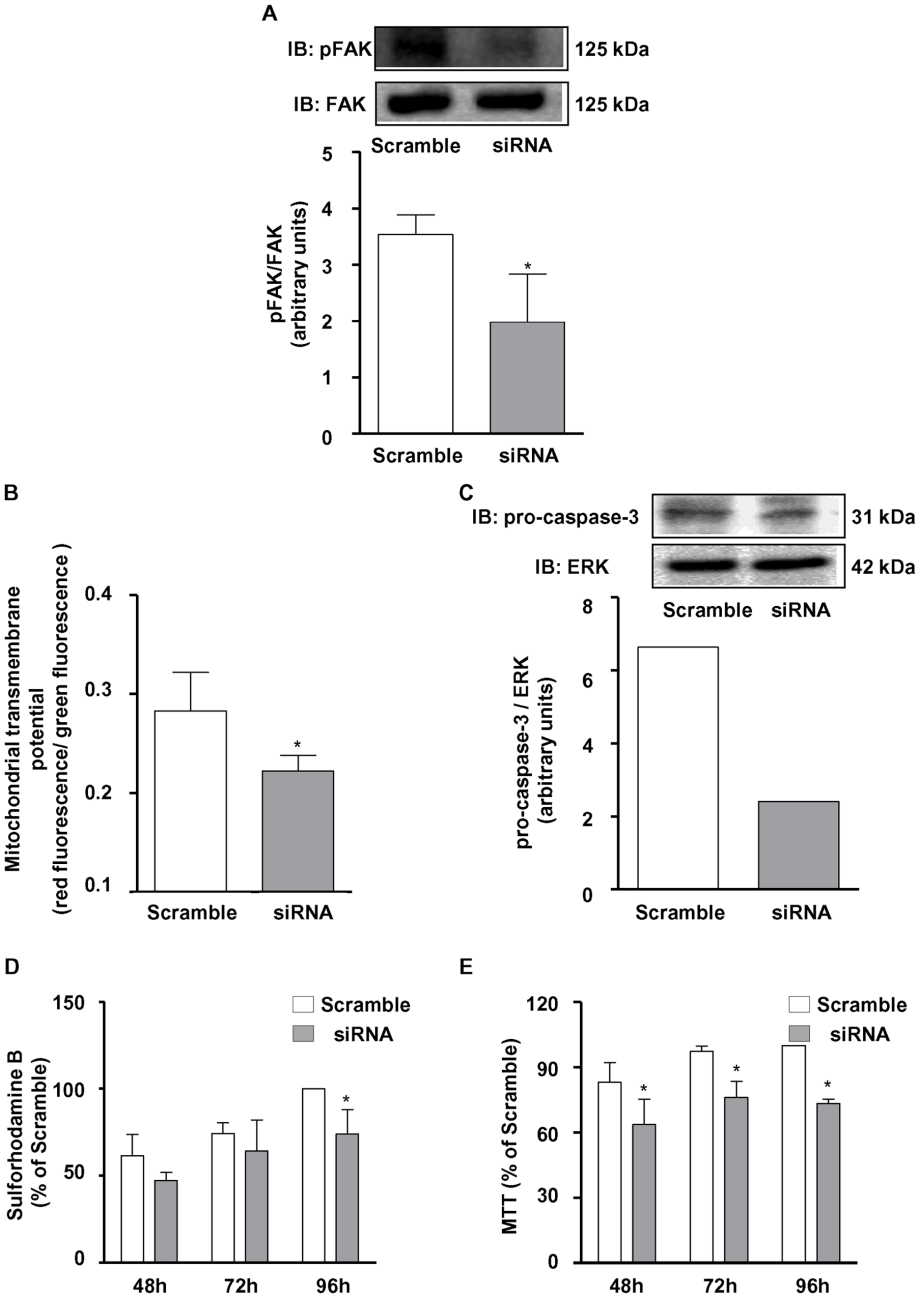
NOX4 silencing reduces MV3 melanoma cell survival and FAK phosphorylation in MV3 melanoma cells. Lysates obtained from transfected cells (10^6^ cells) were subjected to immunoblotting to detect phospho-FAK^Y397^ (A) and caspase-3 (C). (A) Data are expressed as mean ± SD of three independent experiments and images are representative of three independent experiments with similar results. *p<0.05 vs. Scramble. (C) Data shown are representative of three independent experiments with similar results. JC-1 (B), Sulforhodamine-B (D) and MTT (E) assays were performed with MV3 melanoma cells transfected with Sc or siRNA as described in Materials and Methods and the results are shown as percentage of Scramble of three independent experiments *p<0.05 vs. Scramble.

In order to confirm the involvement of NOX4 on the afore-mentioned events, MV3 melanoma cells were transiently transfected with NOX4 siRNA. The transfection promoted partial, but a highly significant decrease in NOX4 mRNA (Fig. 7A) and protein levels (Fig. 7B). Interestingly, we observed that the major source of basal ROS is NOX4, since its silencing significantly inhibited ROS accumulation in MV3 (Fig. 7C). Consistent with data obtained using NADPH oxidase pharmacological inhibition with DPI, NOX4 siRNA treatment decreased phosphorylated FAK content (Fig. 8A), as well as, induced the mitochondrial transmembrane potential dissipation (Fig. 8B), decreased pro-caspase 3 levels (Fig. 8C), and inhibition of MV3 survival at later time points (Fig. 8D, E).

ROS can lead to a down-regulation of protein tyrosine phosphatases, indicating that the redox status of the intracellular environment may have major implications in cell signaling and fate [36]. In order to evaluate whether the effect of NADPH oxidase inhibition on melanoma viability relies on increased protein tyrosine phosphatase activity, MV3 cells were incubated with the protein tyrosine phosphatase inhibitor, Na_3_VO_4_, in the presence or in the absence of DPI. Na_3_VO_4_ had no impact on melanoma viability *per se* (data not shown), however, the pre-treatment with low concentrations of this compound prevented DPI-evoked cell death (Fig. S1), suggesting that intracellular ROS are probably interfering on FAK activity/phosphorylation through their effect on phosphatase activity, in MV3 cells.

## Discussion

During the last few years, ROS have emerged as prominent signaling molecules, and seem to play a central role in key intracellular signal transduction pathways involved in a variety of cellular processes [37]. Aberrant ROS signaling may result in physiological and pathological changes, such as impaired or enhanced cell cycle progression and apoptosis [38], [39]. Furthermore, the maintenance of a pro-oxidant intracellular milieu was shown to be closely related to the establishment and development of a variety of cancers [40].

The involvement of ROS in all stages of cancer development was observed in many cell types [41], [42]. The accumulation of ROS may reflect ineffective antioxidant mechanisms and/or a super-activation of ROS-generating systems such as NADPH oxidase [43], [44]. A role for the NADPH oxidase system was reported in a malignant phenotype of prostate cancer cell [45], on the migration of breast cancer cells [46] and epithelial-mesenchymal transition in melanoma cells [22].

In this work, we show that MV3, a highly metastatic human melanoma cell line, also generates ROS in a NADPH oxidase-dependent manner. The inhibition of NADPH oxidase by DPI, a flavoprotein inhibitor that selectively targets NADPH oxidase in the concentration range used in this study, inhibited ROS production, what was followed by reduced cell survival, indicating a pivotal role of ROS produced by NADPH oxidase in MV3 cell survival. There is also strong evidence that DPI, under these experimental conditions, selectively inhibits NADPH oxidase activity, once it had no impact on NO and ONOO^−^ accumulation.

The NOX2 NADPH oxidase is well known as the main isoform responsible for the production of great amounts of ROS by phagocytes, but it is also found in a variety of non-phagocytic cell types, where it is mainly activated in response to agonists [47]. However, NOX4 stands apart from the rest of the family since it appears to be constitutively active, being primarily regulated by its level of expression and addressed as the main source of ROS in a number of melanoma cell lines [14], [26], [48]. The production of ROS by MV3 cells seems to rely mainly on the activity of NOX4, as we observed through NOX4 siRNA. The participation of NOX2 in NADPH oxidase-mediated effect was excluded once like other melanoma cell linage [26], MV3 cells do not express p47phox subunit (critical to NOX2-containing NADPH oxidase activation), and apocynin did not affect melanoma viability. On the other hand, MV3 cells express high levels of NOX4, which does not require coupling to any cytosolic subunit to be active.

Although the importance of NADPH oxidase-mediated signaling has been demonstrated in different malignant cells, the molecular targets of their products have not been fully elucidated. Previous works have suggested that the effects of the endogenous ROS would rely exclusively on classical pro-survival, redox-sensitive transcription factors, like NF-κB and AP-1 [26]. However, monitoring MV3 cell cultures treated with DPI since early time points (30 minutes) unveiled severe morphological changes that preceded any alteration in cell viability. Those changes provided a valuable clue, leading us to investigate signaling pathways involved in cell adhesion.

Focal adhesions are points of interaction between integrins and extracellular matrix (ECM) and draw together adhesion receptors, as well as signaling and cytoskeletal proteins. They are critical to maintaining cellular shape, survival, growth and migration [49]. The focal adhesion kinase is primarily activated during integrin-mediated cell adhesion to ECM and to a lesser extent by growth factors, bioactive lipids, neuropeptides, and ROS [35]. Autophosphorylation of FAK at Tyr397 residue induces its accumulation to focal adhesion complexes and establishes a close connection between integrins to actin cytoskeleton [50].

When MV3 melanoma cells are seeded on culture dishes, they form a monolayer firmly attached to the substrate, displaying focal adhesion points along the cell edge. However, NADPH oxidase inhibition promoted reduction in cellular spreading, collapsing focal adhesions cortical organization. Moreover, we observed the formation of cortical polymerized actin ring, an indicative of cell detachment.

As a key molecule in the transduction of integrin-mediated signaling, FAK is critically involved in the development and progression of cancer, regulating survival, proliferation, migration and invasion [51]. Not surprisingly, highly aggressive melanoma cell lines contained constitutive high levels of phosphorylated FAK whereas the poorly aggressive melanoma cell lines did not [52]. MV3 melanoma cell line is characterized as highly metastatic [28] and our results confirmed that this phenotype is linked to constitutive high levels of FAK phosphorylated on tyrosine 397.

The phosphorylation of FAK at Tyr397 creates a high-affinity binding site for the tyrosine kinase cSrc, which is then activated [53]. The FAK-Src complex mediates cell migration [54], proliferation [55] and survival [56]. Our data shows that high levels of FAK are constitutively associated to Src in MV3 melanoma cells. However, the inhibition of NADPH oxidase activity by DPI or NOX4 silencing significantly reduced FAK^Y397^ phosphorylation and probably disrupted FAK-Src association. These results indicate once more that ROS derived from a NADPH oxidase, most likely NOX4, seems to modulate focal adhesion dynamics in MV3 cells. Supporting this premise, it has been reported a close relationship between NOX4 activity and focal adhesion formation, involving a novel p22phox binding partner (Poldip2), which stabilizes NOX4-p22phox complexes and increases NOX4 association to focal adhesions [57].

The ROS-induced protein tyrosine phosphorylation seems to rely on their ability to promote post-translational modification on tyrosine kinases and PTP, resulting in their activation and inactivation, respectively [35]. It has also been reported that oxidative inhibition of PTP is a critical step to FAK-mediated cell adhesion [34]. We observed that Na_3_VO_4_, a broad-spectrum PTP inhibitor, totally abolished DPI effect on cell growth (Fig. S1), suggesting that oxidation of PTPs by NADPH oxidase-derived ROS leads to impairment of FAK dephosphorylation and consequent maintenance of FAK-mediated signaling.

Focal contacts disorganization results in a specific form of apoptosis known as anoikis [58]. Studies have showed that resistance to anoikis seems to be involved in the onset and evolution of tumors, as well as in cell growth and metastatic potential [59]. FAK phosphorylation suppresses this specific form of apoptosis [60], whereas attenuation of FAK expression or inhibition of FAK increases apoptosis and suppresses metastasis in tumor cells [61], [62]. Furthermore, other studies have shown ROS involvement in anoikis resistance [63]–[65]. In this study, we noted that DPI-induced cell death seems to involve a significant increase in the hypodiploid population and caspase-3 activation, phenomena that follow a rapid alteration on focal adhesion dynamics, strongly suggesting apoptotic death. Corroborating the NOX4 relevance in MV3 melanoma cell survival, we also observed that NOX4 siRNA induced mitochondrial transmembrane potential dissipation, an early hallmark of apoptosis. Complementary experiments are needed in order to fully characterize the observed cell death as anoikis. We also investigated DPI-induced modulation of mitochondrial-derived ROS. DPI did not affect constitutive mitochondrial ROS production, as evaluated using the specific probe MitoSox (Fig. S2).

**Figure 9:**
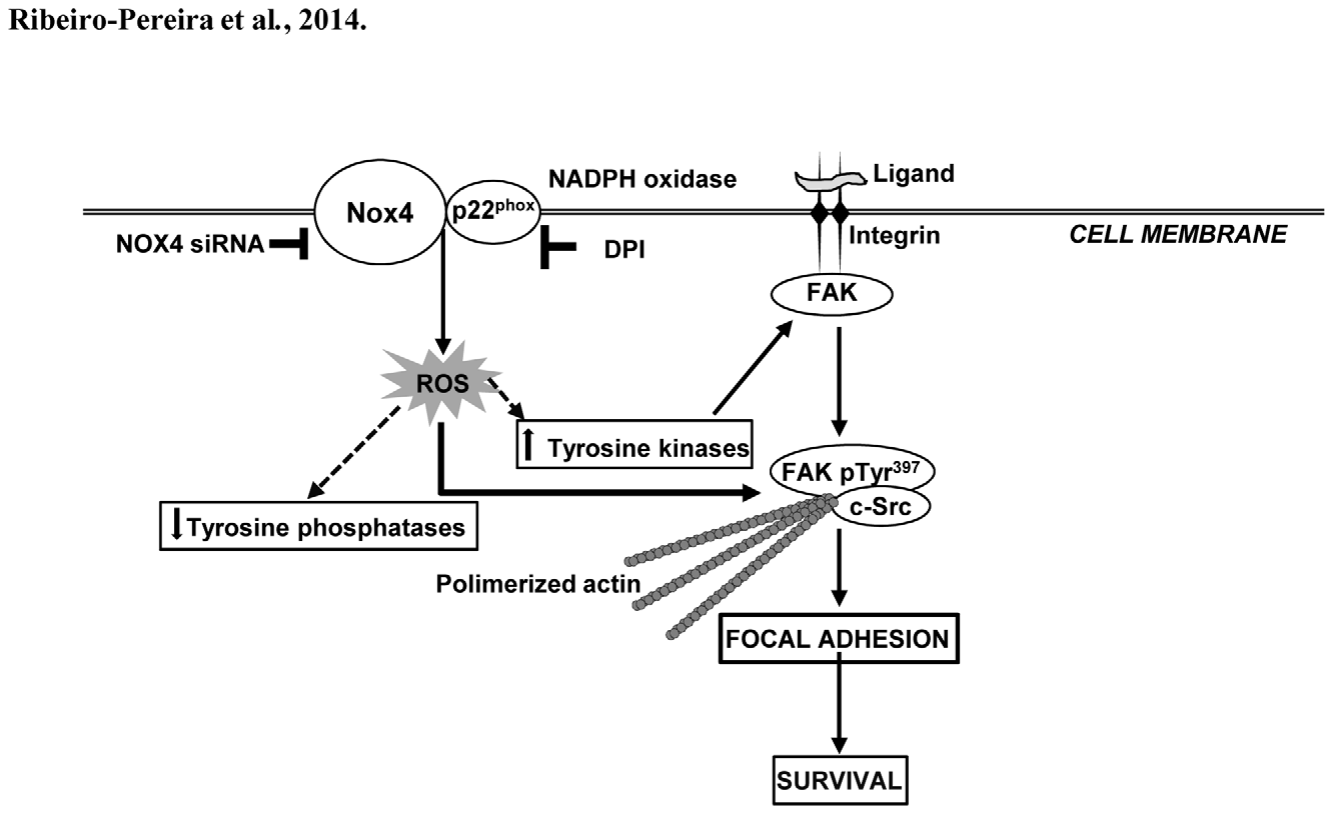
A schematic model for signaling pathway activated by NADPH oxidase-generated ROS on human melanoma cells. NADPH oxidase-derived ROS down-modulate protein tyrosine phosphatases, consequently increasing FAK phosphorylation. FAK^Y397^ phosphorylation creates a high-affinity site for cSrc, as well as stimulates actin polymerization and focal adhesion stabilization, positively modulating cell motility, proliferation and survival. NADPH oxidase inhibition by DPI or NOX4 siRNA reduces ROS production, what probably leads to an increased protein tyrosine phosphatase activity, which, in turn, could inhibit focal adhesion formation/stabilization and induce MV3 human melanoma cells death.

Taken together, our data strongly suggest that NADPH oxidase-derived ROS convey cell survival signals in MV3 melanoma cells through the persistent activation of the FAK pathway, probably inhibiting protein tyrosine phosphatase activity. Our study addresses, for the first time, FAK as an important target of ROS-mediated signaling in melanoma cells, showing that FAK phosphorylation and downstream events are highly sensitive to NADPH oxidase-derived ROS. NADPH oxidase inhibition promotes focal adhesions breakdown and cell death, (Fig. 9). These findings shed light on a still underappreciated face of ROS signaling in cancer cells and may corroborate to the development of more selective and effective strategies in order to control melanoma growth and metastatic colonization.

## Supporting Information

Figure S1 Inhibition of tyrosine phosphatase activity reverts DPI effect on melanoma survival. Cells (6×10^3^) were preincubated for 30 min in the presence or absence of increasing concentrations of Na3VO4 (0.1–3 µM) and subsequently treated with DPI (10 µM) for 48 hours. MTT assay was performed as described. Results are shown as percentage of control and are expressed as mean ± SD of three independent experiments performed in quintuplicate. *p<0.05 vs. control, **p<0.05 vs. DPI.
(TIF)

Figure S2 Inhibition of NADPH oxidase activity does not abolish constitutive mitochondrial ROS generation on melanoma cells MV3. MV3 cells were incubated with or without DPI (10 µM). Mitochondrial ROS production was measured by MitoSox probe oxidation. Data are expressed as mean ± SD of six independent experiments. * p<0.05 vs. control.
(TIF)
