# Supplementary figures and images for: Redox Modulation of FAK Controls Melanoma Survival - Role of NOX4
Source: PLoS One. 2014 Jun 9;9(6):e99481. doi: 10.1371/journal.pone.0099481 (PMC4050056; doi:10.1371/journal.pone.0099481)

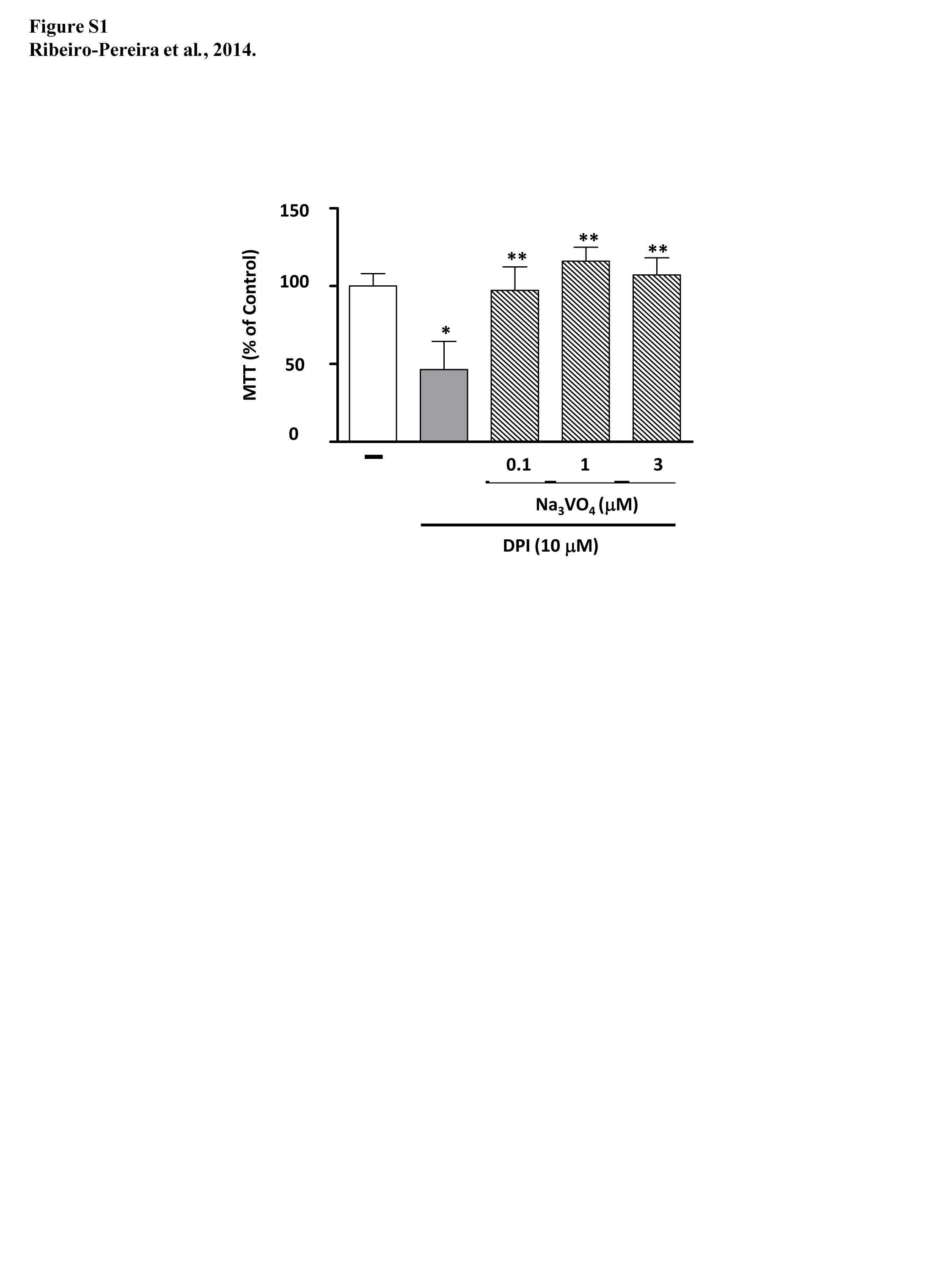

Supplement: Figure S1 — Inhibition of tyrosine phosphatase activity reverts DPI effect on melanoma survival. Cells (6×10^3^) were preincubated for 30 min in the presence or absence of increasing concentrations of Na3VO4 (0.1–3 µM) and subsequently treated with DPI (10 µM) for 48 hours. MTT assay was performed as described. Results are shown as percentage of control and are expressed as mean ± SD of three independent experiments performed in quintuplicate. *p<0.05 vs. control, **p<0.05 vs. DPI. (TIF) [file pone.0099481.s001.tif]

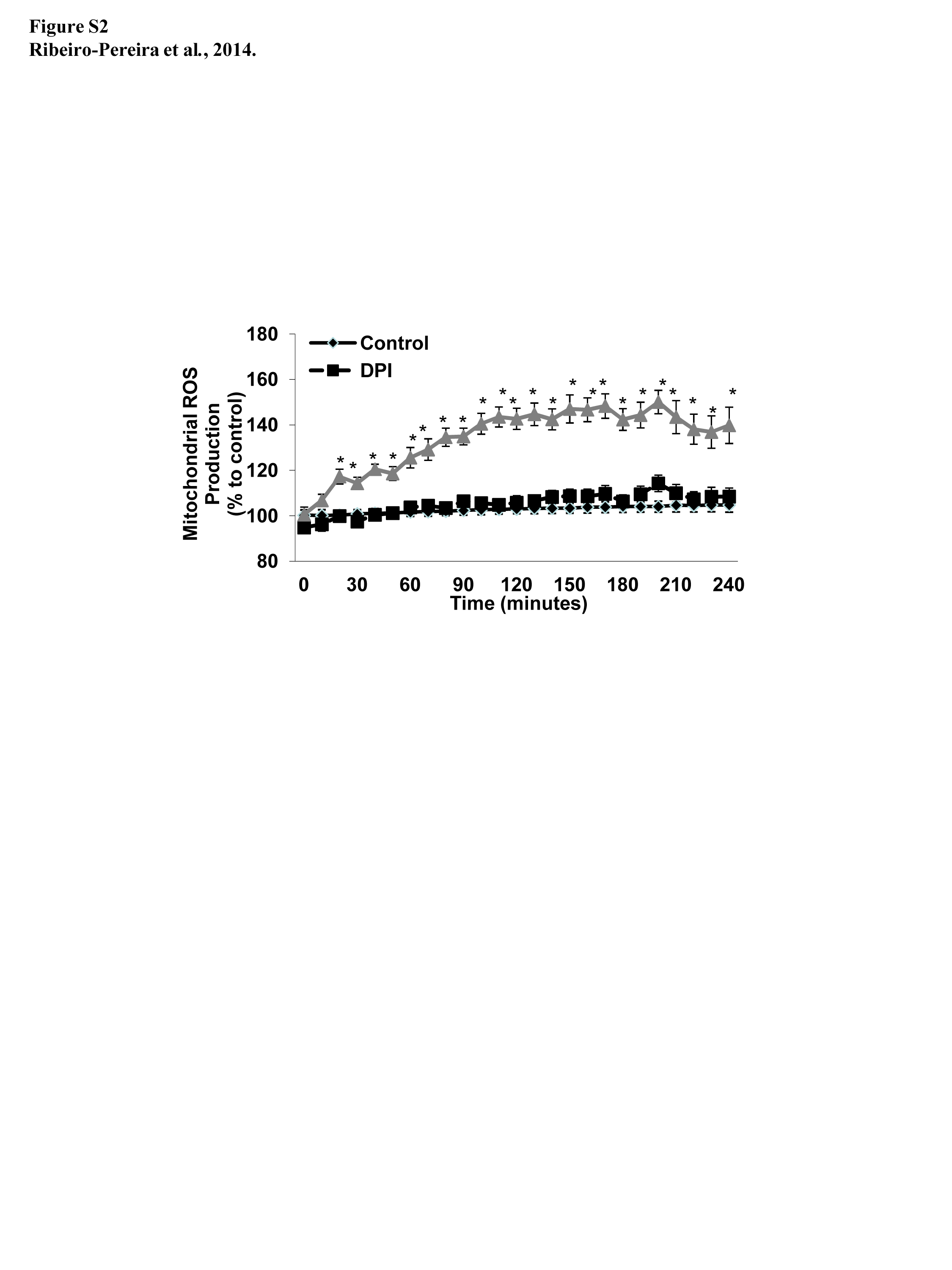

Supplement: Figure S2 — Inhibition of NADPH oxidase activity does not abolish constitutive mitochondrial ROS generation on melanoma cells MV3. MV3 cells were incubated with or without DPI (10 µM). Mitochondrial ROS production was measured by MitoSox probe oxidation. Data are expressed as mean ± SD of six independent experiments. * p<0.05 vs. control. (TIF) [file pone.0099481.s002.tif]
